# Assessment of *In Vitro* and *In Vivo* Bioremediation Potentials of Orally Supplemented Free and Microencapsulated *Lactobacillus acidophilus* KLDS Strains to Mitigate the Chronic Lead Toxicity

**DOI:** 10.3389/fbioe.2021.698349

**Published:** 2021-11-02

**Authors:** Zafarullah Muhammad, Rabia Ramzan, Ruifen Zhang, Dong Zhao, Mehak Gul, Lihong Dong, Mingwei Zhang

**Affiliations:** ^1^ Key Laboratory of Functional Foods, Ministry of Agriculture and Rural Affairs/Guangdong Key Laboratory of Agricultural Products Processing, Sericultural & Agri-Food Research Institute, Guangdong Academy of Agricultural Sciences, Guangzhou, China; ^2^ College of Food Science and Technology, Huazhong Agricultural University, Wuhan, China; ^3^ Shaikh Khalifa Bin Zayed Al-Nahyan Medical & Dental College, Lahore, Pakistan

**Keywords:** probiotics, *L. acidophilus*, maize resistant starch, microencapsulation (MC), oral supplementation, Pb toxicity, *in vitro* kinetic modeling, *in vivo* detoxification

## Abstract

Lead (Pb) is a pestilent and relatively nonbiodegradable heavy metal, which causes severe health effects by inducing inflammation and oxidative stress in animal and human tissues. This is because of its significant tolerance and capability to bind Pb (430 mg/L) and thermodynamic fitness to sequester Pb in the Freundlich model (*R* ^2^ = 0.98421) *in vitro*. *Lactobacillus acidophilus* KLDS1.1003 was selected for further *in vivo* study both in free and maize resistant starch (MRS)–based microencapsulated forms to assess its bioremediation aptitude against chronic Pb lethality using adult female BALB/c mice as a model animal. Orally administered free and microencapsulated KLDS 1.1003 provided significant protection by reducing Pb levels in the blood (127.92 ± 5.220 and 101.47 ± 4.142 µg/L), kidneys (19.86 ± 0.810 and 18.02 ± 0.735 µg/g), and liver (7.27 ± 0.296 and 6.42 ± 0.262 µg/g). MRS-microencapsulated KLDS 1.0344 improved the antioxidant index and inhibited changes in blood and serum enzyme concentrations and relieved the Pb-induced renal and hepatic pathological damages. SEM and EDS microscopy showed that the Pb covered the surfaces of cells and was chiefly bound due to the involvement of the carbon and oxygen elements. Similarly, FTIR showed that the amino, amide, phosphoryl, carboxyl, and hydroxyl functional groups of bacteria and MRS were mainly involved in Pb biosorption. Based on these findings, free and microencapsulated *L. acidophilus* KLDS 1.0344 could be considered a potential dietetic stratagem in alleviating chronic Pb toxicity.

## Introduction

The ever-increasing population and extensive utilization of conventional and nonconventional sources pollute the environment and pose a severe threat to human health (Kumar et al., 2020). Heavy metals are continuously being accumulated in the atmosphere, especially in food and water, due to industrial expansion (Ali and Khan, 2019; Wu et al., 2021), smelting, synthetic compounds production, mining operations, inappropriate waste disposal, agricultural manure (Gomiero, 2018), pesticides, airplanes, cosmetics, hair dyes, leaded gasoline of vehicles (Singh Sankhla et al., 2021), ceramics, water pipes, solders, Ayurvedic drugs, and canning and packaging of food materials and equipment (Sun et al., 2021). The provoked lethality in all living species is owing to the non-biodegradability and accumulation of these metals into the food chain (Kang et al., 2016). Altered biochemical and physiological characteristics, and oxidative stress are the concerned mechanisms of heavy metal toxicity in humans (Nicolás-Méndez et al., 2020). According to the recent epidemiological shreds of evidence, the mounting burden of autoimmune and metabolic diseases related to respiration and infant infections on a global scale is thought to be because of heavy metal pollutants (Feng et al., 2020).

Lead (Pb) is a heavy metal that is persistently available in the earth’s crust, water, and air (Kabeer et al., 2019; Kumar et al., 2020). It possesses unique properties of low melting point, ductility, softness, and high malleability, due to which it is frequently used in the operations as mentioned above (Ketsela et al., 2020). Pb is a nonbiodegradable and non-essential heavy metal with 30–35 days of biological half-life in the blood, but it can remain in the skeletal system and brain for years (Maret, 2017; Rodríguez and Mandalunis, 2018). These properties make it a potent heavy metal toxic contaminant, grievously threatening the human health (Specht et al., 2016). According to the IARC (International Agency for Research on Cancer), ATSDR (Agency for Toxic Substances and Disease Registry), and WHO (World Health Organization), lead is the second most hazardous carcinogen in the human population, imposing acute to chronic toxicity even at minute concentrations (Kenny et al., 2020).

The main routes of human exposure to lead are generally gastrointestinal and respiratory tracts and, to some extent, skin (leaded gasoline) (Li L. et al., 2017; Kenny et al., 2020). Depending on the age (faster in young children) and nutritional factors (Ca, Zn, Fe, Mg deficiency), the deposition of lead takes place in the lungs through the respiratory tract, liver, kidneys, bones, and then enter into systematic circulation (Olawoyin et al., 2018). Imbalanced calcium homeostasis, carcinogenesis, degenerative variations, tissue illnesses, hematological, cardiovascular (Boskabady et al., 2018), renal, reproductive, skeletal, respiratory, and irreversible neurological disorders in kids are the common disorders associated with Pb toxicity (Taylor and Schniering, 2010; Boskabady et al., 2018). The cell homeostasis is disturbed when Pb ions replace several bivalent and monovalent cations like Mg^2+^, Ca^2+^, Fe^2+^, and Na^+^, which finally disrupts inflammatory responses, antioxidant mechanisms, and enzyme regulations resulting in oxidative stress (Muhammad et al., 2018). Oxidative stress is caused by Pb through the production of reactive oxygen species. These ROS are produced by the disturbed metabolisms of bile acids, energy, amino acids, and vitamin E (Liu et al., 2019). The antioxidant defense system is weakened by the depletion of glutathione which affects the functioning of GPX (glutathione peroxidase), SOD (superoxide dismutase), and increases the oxidation of DNA, proteins, and lipids. So, oxidative stress damages the cell membrane and plays an essential role in imparting adverse health effects through Pb-induced toxicity (Chedea et al., 2019; Liu et al., 2019).

Commonly, CaNa2EDTA (calcium disodium versenate) and DMSA (meso-2,3-dimercaptosuccinic acid) are used as chelating agents to treat the Pb toxicity, and this method is termed as chelating therapy (Li B. et al., 2017; Muhammad et al., 2018). These agents are administered into the body intraperitoneally or subcutaneously, which can precipitate, causing severe side effects. Similarly, these agents relatively lack selectivity. These also chelate the trace elements (Zn, Fe, Mg, and Cu) that are necessary for the antioxidant system imposing subsequent deficiency of these trace elements (Bayer, 2015; Wei et al., 2019). Likewise, due to efficacy and safety issues, these agents can cause renal toxicity, anorexia, appetite loss, nausea, vomiting, malaise, and skin reactions. High doses and long-term treatment of these agents are not suitable for chronic Pb toxicity (Shaban et al., 2021). These reasons necessitate the hunting of alternative, natural, efficient, safe, and economical dietary compounds to counteract the chronic Pb toxicity.

Using microorganisms alone or in combination with natural polymeric compounds to recover and remove the heavy metal cations is termed as biosorption or bioquenching, which could be a potential technique alternative to chelating therapy (Ubando et al., 2021). Because it is claimed to be a promisingly efficient, safe, and economical method compared to the adsorption, ion exchange coagulation, and chelating remediation methods (Beni and Esmaeili, 2020). Some agricultural-based polymeric substances are getting attention as suitable sorption matrices (Baharuddin et al., 2019). Some recent studies have validated their capacity to sequester Pb levels in tissues and blood to decrease oxidative stress (Jafarpour et al., 2017). Being the most abundant, multifunctional, biocompatible, biodegradable, and economical biopolymer, starch has gained expansive use in the food and pharmaceutical industry (Baharuddin et al., 2019; Priyan et al., 2020). Maize-resistant starch (MRS) is considered to have a compact structure and high amylopectin contents with more excellent resistance to enzymatic hydrolysis (Muhammad et al., 2021). The properties of having greater amylopectin content, surface area, and Werner-type complexes have increased the ability of maize-resistant starch to ligate metal cations with hydroxyl groups of its D-glucose units (Kirn and Lim, 1999; Hoque et al., 2019). Bhat et al. conducted a study to assess the efficiency of potato-resistant starch (PRS) against heavy metals and stated that it could be an effective adsorbent with 78.1% efficiency against Pb adsorption (Bhat et al., 2015).

As far as the functional food industry is concerned, safety, quality, and feasibility cannot be compromised while selecting biosorbents (Bhattacharya, 2019; Duan et al., 2020). Due to having specificity, low cost, and environmentally friendly and GRAS status, lactic acid bacteria are considered superorganisms to detoxify the dietary toxins for bioquenching (Zanjani et al., 2017; Allam et al., 2018). Several studies revealed that *L. crispatus* and *L. acidophilus* showed significant activity against heavy metals by producing S-layer proteins (Schär-Zammaretti and Ubbink, 2003; Ubbink and Schär-Zammaretti, 2007). Zhai et al. studied the protective effects of *L. plantarum* to deal with Cd-induced oxidative stress. The results showed a significant reduction in Cd absorption, accumulation in the intestine, blood and tissues (Zhai et al., 2017). According to Elsanhoty et al., *L. acidophilus* bacteria were found to be more effective in removing the Pb ions. Some studies revealed that LAB has specific membrane structures where the metal ions are passively bound and removed from the body (Elsanhoty et al., 2016; Ojekunle et al., 2017). According to Zhai et al., Li et al., and Muhammad et al., orally administered microencapsulated and nonencapsulated LAB showed healing effects against Pb-induced oxidative stress and decreased the levels of Pb from blood and tissues (Zhai et al., 2015; Li B. et al., 2017; Muhammad et al., 2018). Similarly, Al-Wabel et al. and Jafarpour et al. found an effective role of LAB in increasing the activity of anti-oxidative enzymes to protect the liver and renal tissues (Al-Wabel et al., 2007; Jafarpour et al., 2017). According to the WHO requirements, probiotics 10^6^–10^7^ CFU/ml viable bacteria are needed to yield their therapeutic functions on consumption. Microencapsulating these bacteria with natural polysaccharides is the ultimate approach to retain their viability in harsh processing and gastrointestinal conditions (Muhammad et al., 2017, 2018, 2019).

In our recently published study (Muhammad et al., 2021), we integrated resistant starches for the microencapsulation of *L. acidophilus* KLDS 1.1003 and selected maize resistant starch (MRS) microencapsulated bacteria for the present research work. Due to the insufficient availability of natural remedies to cope with the Pb-induced chronic toxicity and adverse effects of chelating agents, there is an urgent need to develop natural strategies based on natural ingredients. Currently, no study is available on the symbiotic therapeutic potential of orally supplemented *L. acidophilus* 1.1003 microencapsulated with maize resistant starch to deal with chronic lead toxicity. In the present work, an appraisal of Pb alleviation and biosorption potentials of MRS-microencapsulated and nonencapsulated or free *L. acidophilus* KLDS 1.1003 has been conducted. Additionally, their symbiotic protective effects on (enzymatic and non-enzymatic) anti-oxidative responses and mechanisms, reduction in intestinal Pb absorption, Pb tolerance, Pb deposition in tissues, and reduction of oxidative stress in hepatic and renal tissues were appraised. Overall, the basic objectives of the present research work were the *in vitro* and *in vivo* evaluation of the combined symbiotic protective role and the bioremedial therapeutic role of orally supplemented MRS based microencapsulated and free *L. acidophilus* KLDS strains of bacteria against induced oxidative stress due to chronic exposure to Pb (lead) by using the adult female BALB/c mice as a model animal.

## Materials and Methods

### Chemicals and Material Components

The kit used to determine the Pb levels from the whole blood samples was (BH 2100 kit). It was purchased from Beijing Bohui Innovation Technology Co., Ltd. All other kits were acquired from Nanjing Jiancheng Bioengineering Institute (Nanjing, China). These kits include Njjcbio C009 which was used to determine ALT (alanine aminotransferase), and Njjcbio C010 was used to determine AST (aspartate aminotransferase). Similarly, Njjcbio A001 and Njjcbio A003 kits were used to determine SOD (superoxide dismutase) and MDH (malondialdehyde), respectively. Likewise, Njjcbio A005 was used to determine the GSH–Px (GSH peroxidase), and Njjcbio A006 was used for GSH (glutathione). And Njjcbio A007 kit was used to determine CAT (catalase) activity. *δ*-Aminolevulinic acid and maize-resistant starch (CAS: 9005-25-8) were procured from Sigma Aldrich (Mainland, China). Lead nitrate and all other reagents used in the experiments were of analytical grade and procured from Jinan Boss Chemical Company (Jinan, China).

### Cultures and Bacterial Strains

*L. acidophilus* KLDS strains (KLDS 1.0901, 1.902, 1.1003, AD_3_, L_2_ and L_6_) were isolated from the traditional yogurt products in the Inner Mongolian region of China. After 16S rRNA gene similarity and API 50CH strip analyses, these strains were stored at the KLDS laboratory administered by the Ministry of Education, China. Reactivation of the frozen stock culture of *L. acidophilus* strains was done two times in de Man, Rogosa and Sharpe agar or the MRS broth, incubation of bacterial strains was carried out at 37°C until the stationary phase was reached. Then, the collection of cell pellets was done by centrifuging samples at 10,000× g for 10 min at 4°C and using a 21 micro centrifuge (Thermo Sorvall Legend Micro). Sterilized distilled water was used for pellet suspension, but before the suspension, the pellets were washed thrice.

### Pb Tolerance and Binding Capacity Assessment of Bacterial Strains

The tolerance and binding capability of six *L. acidophilus* strains (KLDS 1.0901, 1.902, 1.1003, AD_3_, L_2,_ and L_6_) were carried out using a minutely modified and previously described method by Li B. et al. (2017). The biomass was cultivated after 16 h of incubation and centrifugation for 10 min at 4°C and 10,000× g. The centrifuged biomass was washed twice with purified water, and the cell pellets were collected afterward. Then, 100 mg/L of lead nitrate was taken in distilled water, and a bacterial concentration (wet weight) was set to 1 g/L in the same water. Later, the bacterial mass and lead nitrate mixed solution was incubated at 37°C for the period of 24 h. After incubation, the samples were centrifuged at 10,000 g for 20 min. After subsequent centrifugation, the supernatants were analyzed to determine the residual Pb concentrations in the supernatants. A flame atomic absorption spectrophotometer (Spectra AA 220; Varian, Palo Alto, CA, United States) was used for the purpose, and the metal removal efficiency was calculated with the following equation (Eq. 1).

$$\mathrm{Removal}\left(\text{\%}\right)=\;\frac{C_{i\;-}\;C_{e}}{C_{i}}\times 100$$ (1)

Here, C_*e*_ represents the post-removal residual Pb concentration and C_*i*_ denotes the post-removal initial Pb concentration. The lead (Pb) tolerance of each strain was determined by implicating the minimum inhibitory concentration approach or MIC as described by Gupta et al. (2012) and Zhai et al. (2015). First of all, the MRS agar medium was prepared with the lead nitrate (100–1,000 mg/L) solution. The cultured LAB strains (10 µL) were spread separately on the MRS agar medium by taking the 1 × 10^9^ CFU/ml inoculum. Later on, the inoculated plates were incubated at 37°C for 48 h and determined the LAB strains growth. The minimum Pb concentration, which entirely repressed the growth of LAB strains, was taken as the MIC for the present research work.

### Equilibrium Isotherm

The equilibrium isotherm study was carried out by following the method described by Chakarvarty et al. (Chakravarty and Banerjee, 2012). According to this method, lead nitrate (5–60 mg/L) containing ultrapure water was used to suspend the harvested cell pellets and dry weight (1 g/L)-based final bacterial concentration was prepared. The initial pH for the Pb binding assay was taken as 6.0, and the bacterium bound Pb equilibrium content was determined by the following Eq. 2.

$$Q_{e}\left(\mathrm{mg metal}/\mathrm{g biosorbent}\right)=\;\frac{Ci—Ce}{m/V}$$ (2)

where C_*i*_ denotes the post-removal initial Pb concentration, and C_*e*_ represents the post-removal residual Pb concentration, respectively, whereas m/V = 1 g/L. The determination of sorption equilibrium between the metal ions and biosorbents was carried out using the Langmuir and Freundlich models, and nonlinear regression methods were used to obtain isotherm constants for the models (Khayyun and Mseer, 2019).

### Microencapsulation of Bacterial Strain, Survival After *In Vitro* Digestion and Storage

*L. acidophilus* KLDS 1.1003 were microencapsulated by using maize-resistant starch-based formulations. And the process of spray-dried microencapsulation was carried out using Buchi B-290 (Flawil, Switzerland) lab-scale spray dryer. The appraisal of probiotic’s survival during *in vitro* digestion and storage was done following the previously reported method in our formerly published research article (Muhammad et al., 2017, 2018). According to our previous study, maize-resistant starch (MRS) based formulations provided the best protection to probiotic strains during spray-dried microencapsulation, *in vitro* digestion, and storage. Similarly, *L. acidophilus* KLDS 1.1003 has shown highest Pb binding capability as compared to other KLDS strains, so MRS-based microencapsulated KLDS 1.1003 was selected as the best suitable strain for mice model experiments. The microencapsulated bacterial strains were lyophilized by mixing with skimmed milk and stored at −20°C. Skimmed milk acts as a cryoprotectant agent and protects lyophilized bacterial cells against below freezing temperature. The viability of the freeze-dried bacterial cells was done by colony counting, before conducting the animal model experiments. Briefly, the reactivation of freeze-dried bacterial cells by using distilled water was carried out. The documented bacterial cells viability was calculated as 2.7 × 10^9^ CFU/ml. During the experimentation period, the reactivated lyophilized probiotic bacteria were mixed in skimmed milk through oral gavage to each mice by taking the oral dose of 0.5 ml conforming to almost 1.3 × 10^9^ CFU of microencapsulated KLDS 1.1003.

### Scanning Electron Microscopy Analyses of Untreated and Pb Treated Bacteria

The preparation of SEM samples was done by following the method described by Wang et al. (2017). 2.5% glutaraldehyde (v/v) was used to fix the harvested cell pellets, which were Pb treated (60 mg/L) and untreated. This fixing process was carried out at 4°C for 1.5 h, and the pellets were washed with phosphate buffer solution three times. After discarding the supernatants, the treatment of cell pellets with alcohol was done. A mixture of t-butanol and alcohol (1:1) with 50, 70, 90, and 100% alcohol concentrations were used for successive washing of cell pellets. Lastly, the elution of cell pellets was done with plain t-butanol. After elution, the samples were freeze-dried for 4 h and then sputter coated with gold. The scanned micrographic photographs were taken by using SEM, coupled with EDS or an energy dispersive spectrometer.

### FT-IR Analyses

FT–IR analyses of the untreated and Pb treated cell pellets were carried to analyze the changes in the functional groups of these treatments. These untreated and Pb treated (60 mg/L) harvested cell pellets were freeze-dried and then mixed with potassium bromide (KBr) powder to prepare KBr discs. These KBr discs contained a finely ground powder (2% w/w) of each sample.

### *In Vivo* Evaluation of Free and Microencapsulated *L. acidophilus* KLDS1.1003 Promises Countering Induced Chronic Pb Toxicity

#### Animal Experiment

Adult female BALB/c mice were obtained from the Huazhong Agricultural University (Animal Center, Wuhan, China). The mice were housed in a well-managed room with controlled temperature (25°C), the environment with standard dark and light cycles (12 h light-dark cycle). Well-designed stainless steel cages were used to keep the mice, and a free supply of commercially available standard mice food was given throughout the trial. The *ad libitum* sterilized water supply was also managed, and Pb free and Pb contaminated water was supplied to the therapy groups. The approved protocols by the Ethics Committee of Guangdong Academy of Agricultural Sciences and Guangdong provincial Animal Care Committee, Guangzhou, China, were strictly followed during the trial. Also, the methods used during the study were followed in accordance with the recommended guidelines of the European Community to handle the animals that are used for experimental trials (directive 2010/63/EU). The mice were kept for 1 week without giving any treatment to adapt to the environmental conditions.

The group division of mice is given in Table 1. Six subgroups of mice were taken, with ten mice in each group as follows: C +ve (control with no Pb or KLDS 1.1003), C −ve (Pb only), free *Lactobacillus acidophilus* KLDS 1.1003 only, MRS based microencapsulated *Lactobacillus acidophilus* KLDS 1.1003 only, Pb plus free *Lactobacillus acidophilus* KLDS 1.1003, and Pb plus encapsulated *Lactobacillus acidophilus* KLDS 1.1003. The oral dose of 1.3 × 10^9^ CFU of *Lactobacillus acidophilus* KLDS 1.1003 mixed in 0.5 ml of skimmed milk was gavage fed to the mice of therapy groups. The oral dose was given once a day/every day during the trial period. The oral dose of Pb was prepared by mixing lead nitrate (100 mg/L) in sterilized water. This dose was selected based on the average Pb concentration to which humans are environmentally exposed in Pb polluted areas (Muhammad et al., 2018). This lead-contaminated water was given to the mice groups in order to expose them to the induced chronic lead toxicity. During this trial, the body weights were measured every week.

**TABLE 1:** Protocols for the animal experiment.

	Groups	Feeding plan
Non lead exposed	C +ve	SM+PW
	Free *L. acidophilus* KLDS 1.1003	SM+PW+ KLDS KLDS 1.1003
	MRS-based encapsulated *L. acidophilus* KLDS 1.1003	SM+PW+ encapsulated KLDS 1.1003
Lead exposed	C −ve	SM + Pb (in drinking water)
	Free *L. acidophilus* KLDS 1.1003+lead	SM+ Pb (in drinking water)+KLDS 1.1003
	MRS-based encapsulated *L. acidophilus* KLDS 1.1003+lead	SM+ Pb (in drinking water)+encapsulated KLDS 1.1003

Note: Skimmed milk (SM) daily 0.5 ml through gavage; plain drinking water (PW); Pb (in drinking water), lead nitrate at 100 mg/L (in drinking water); and SM+free and MRS-based encapsulated KLDS 1.1003, 1.4 × 10^9^ CFU *L. acidophilus* KLDS 1.1003 in skimmed milk (SM) daily 0.5 ml through gavage. The experimental treatments lasted for 7 weeks.

Similarly, the fecal samples of each group were also collected weekly. Before fecal collection, the mice were transferred to separate cages for 1 hour. At the end of the 7th week, the individual transfer of mice was carried out in metabolic cages, and animals were kept in these cages for 24 h. After 24 h stay, the blood samples were collected from the eye balls of the mice by using the heparinized tubes to get plasma from these samples. Moreover, redtop plastic tubes were used to collect the blood samples for the serum analyses.

Afterward, the livers and kidneys were removed from the mice bodies and these organs were washed with sterile normal saline solution with a concentration of 0.9% NaCl. The saline solution was simply prepared by mixing sterilized water and NaCl. After washing, the samples were preserved in 10% formalin saline for 2 days and then, the histopathological study was carried out. Extra samples of kidneys and livers were put into cryotubes and stored at −80°C after wrapping them with the aluminum foil in order to post the scarifying assessment of biochemical assays and chemical elements (Hasanein and Emamjomeh, 2019).

#### Assessment of Chemical Elements in Tissues

The measurement of essential metals in the tissue samples was done by digesting them in Omni: CEM (United Kingdom) manufactured metal-free digestion vessels. Concentrated nitric acid was used for sample digestion, and MARS: CEM (United Kingdom) manufactured microwave digestion system was used for the purpose. The metal (Ca, Mg, Zn, and Fe) quantities in the liver and kidney samples were measured. Metal quantification was done with a flame atomic absorption spectrophotometer (AAS) or graphite furnace (Meucci et al., 2020).

#### Quantification of Lead (Pb) in Whole Blood Samples, Liver and Kidney Tissues

The Pb levels in whole blood samples were measured by using a BH 2100 kit. The measurement of essential metals in the tissue samples was done by digesting them in Omni: CEM (United Kingdom) manufactured metal-free digestion vessels. Concentrated nitric acid was used for sample digestion, and MARS: CEM (United Kingdom) manufactured microwave digestion system was used for the purpose. The Pb quantities in the liver and kidney samples were measured. Pb quantification was done with a flame atomic absorption spectrophotometer (Specter. AA; Varian or AAS). The representative units for lead measurement in blood and tissues are given as µg/L and µg/g, respectively (Dobrakowski et al., 2016; Meucci et al., 2020).

#### Biochemical Assays

The kits used for the assays are given in brackets in front of each biochemical assay. These biochemical assays include, GSH-Px (Njjcbio A005), GSH (Njjcbio A006), SOD (Njjcbio A001), and CAT (Njjcbio A007) activities and MDH (Njjcbio A003), GSH (Njjcbio A006) levels in tissues and ALT (Njjcbio C009), AST (Njjcbio C010) activities in serum. These kits were procured from the Nanjing Jiancheng Bioengineering Institute (Nanjing, China), and the guidelines of the kit manufacturing company were followed for all the biochemical assays, and all assays were done thrice (Dobrakowski et al., 2016; Yu et al., 2017).

#### Histopathological Studies

Histopathological studies were carried out after keeping the tissue (liver and kidney) samples for 48 h in 10% formalin. Before analyses, these organs were washed with double distilled (ddH_2_O) or ultrapure water and dehydrated. A rotary microtome was used to cut the paraffin-embedded samples of 5 μm thickness. These 5 μm slices were then stained with H & E (haematoxylin-eosin) dyes, and optical microscopic analyses were done (Li B. et al., 2017; Yu et al., 2017; Muhammad et al., 2018).

### Statistical Analyses

All experiments were performed thrice to compile the data. The statistical analyses were carried out by Statistics 8.1 (Analytical Software, United States), and the Tukey method was used for the comparison of means and the analysis of variance (ANOVA). Statistically, the values of *p* < 0.05 were taken as significant values. All the values are represented as the mean ± SD.

## Results

### Tolerance and Pb Biosorption of Lab Strains

Lead (Pb) binding and tolerance abilities of six *Lactobacillus acidophilus* KLDS strains are given in Table 2. The exhibited Pb removal range of these tested strains was 45.28–76.20% (Table 2), whereas the initial Pb concentration was100 mg/L. *L. acidophilus* KLDS 1.1003 showed the highest Pb binding ability among all the tested strains. Keeping in view of the Pb binding ability of *L. acidophilus* KLDS 1.1003, it was chosen as the best apposite strain to be studied for further *in vivo* analyses.

**TABLE 2:** Pb tolerance and binding ability of the tested *Lactobacillus acidophilus* KLDS strains.

Strains	Lead removal by wet biomass (%)	Minimum inhibitory concentration for Pb (mg/L)
*L. acidophilus* KLDS1.0901	45.28^e^	300.00 ± 2.42^b^
*L. acidophilus* KLDS 1.0902	64.36^b^	170.00 ± 1.73^d^
*L. acidophilus* KLDS 1.1003	76.20^a^	430.00 ± 0.32^a^
*L. acidophilus* KLDS AD_3_	48.37^f^	230.00 ± 0.31^c^
*L. acidophilus* KLDS L_2_	54.61^d^	58.00 ± 0.12^f^
*L. acidophilus* KLDS L_6_	62.00^c^	33.00 ± 5.64^e^

Incubation was done with an initial Pb nitrate concentration of 100 mg/L. Values are mean ± SD of three determinations. Different letters indicate the significant differences (*p* < 0.05) among the strains.

### Evaluation of Biosorption and Isotherms Models

**FIGURE 1:**
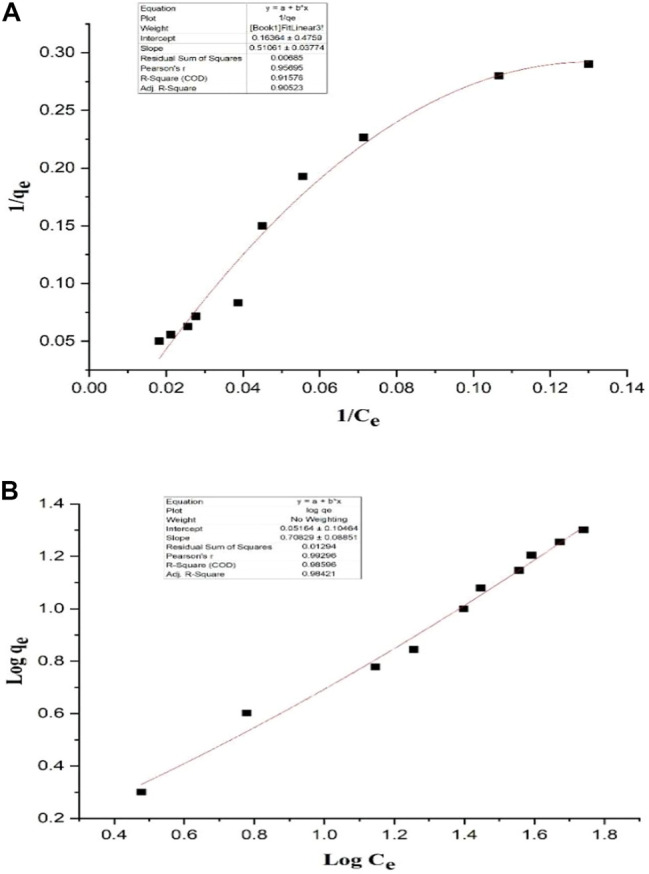
**(A, B)** Adsorption isotherm of Pb binding by *L. acidophilus* KLDS 1.1003.

As shown in Figures 1A,B, the concentration of Pb in the solution has a positive correlation with the Pb-binding ability of *L. acidophilus* KLDS 1.1003. The respective data of different isotherm models, including the Freundnlich and Langmuir models, have been given in Table 3. According to the coefficients of the correlation (*R* ^2^) values, it is clear that the Freundlich model with *R* ^2^ = 0.98421 is the best suitable model for our experimental data.

**TABLE 3:** Adsorption constants derived from simulations with different isotherm models.

Name of isotherm model	Constant	Value
Langmuir isotherm 1/qe = 1/KLqmax*1/Ce+1/qmax	q_max_	6.1124
	K_L_	0.3204
	R_L_	0.0587
	R^2^	0.9052
Freundlich isotherm Logqe = Log Kf+1/nLogCe	1/n	1.4118
	K_f_	25.8134
	R^2^	0.9842

q_e_ (mg metal/g biosorbent) represents the equilibrium content of the Pb bound by the biomass.

C_e_ (mg/L) represents the equilibrium Pb concentration.

**FIGURE 2:**
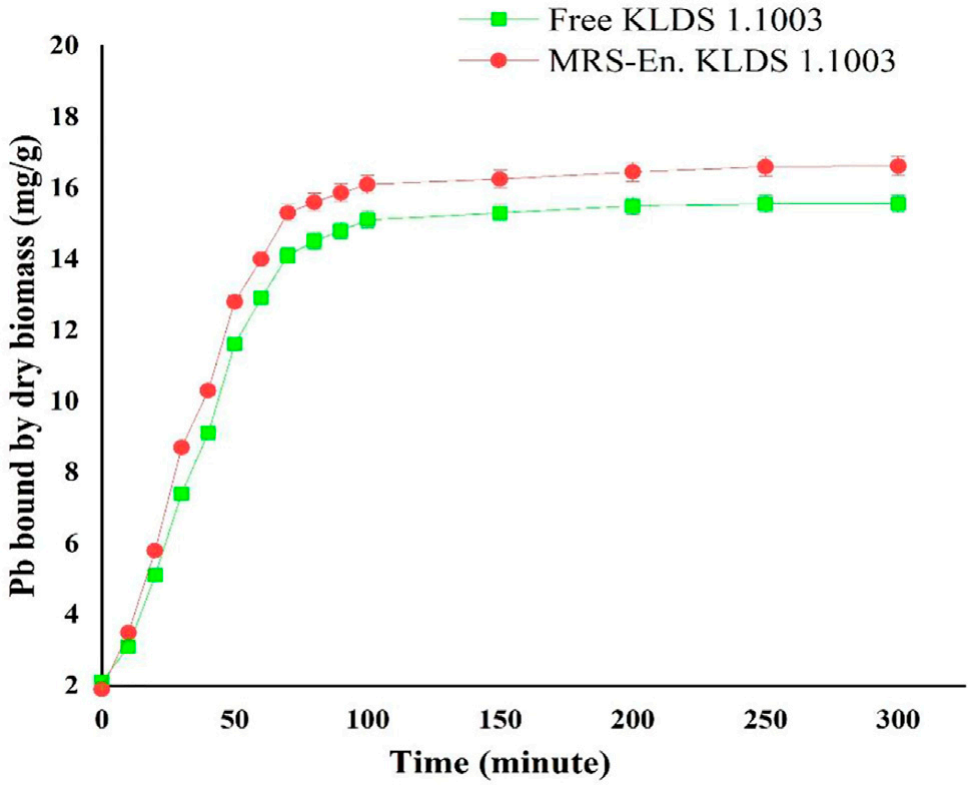
Lead binding of free and MRS-based encapsulated *L. acidophilus* 1.1003 at different time points. Values are mean ± standard deviation (SD).

In addition, maize-resistant starch (MRS) based microencapsulated *L. acidophilus* KLDS 1.1003 also indicated the more lead binding capacity followed by free bacteria (Figure 2), and the completion of the binding process was taken place in about 80 min.

### Electron Microscopy Analysis

**FIGURE 3:**
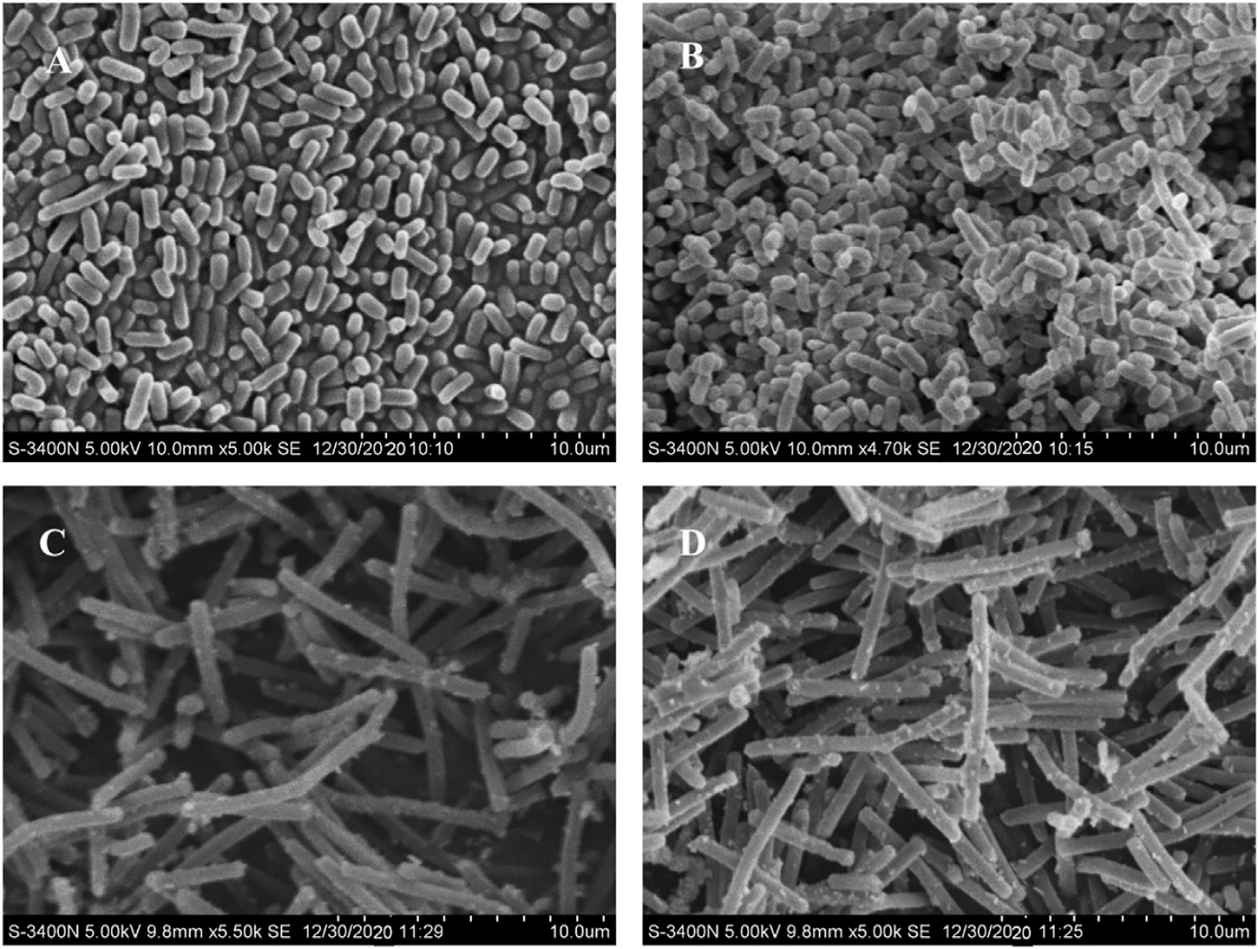
Scanning electron microscopy (SEM) micrographs of free and MRS-based encapsulated *L. acidophilus* KLDS 1.1003 untreated and treated with Pb (60 mg/L). **(A)** *L. acidophilus* KLDS 1.1003 only, **(B)** MRS-based encapsulated *L. acidophilus* KLDS 1.1003 only, **(C)** *L. acidophilus* KLDS 1.1003 plus lead, **(D)** MRS-based encapsulated *L. acidophilus* KLDS 1.1003 plus lead.

After the lead (Pb) treatment, the SEM micrographs of free and MRS-microencapsulated *L. acidophilus* KLDS 1.1003 indicated that the various light clusters of metal precipitates were present, but these clusters did not show the even coverage of the cell surface (Figures 3C,D). Conversely, the light precipitates were not found on the cell surface of the untreated pellets (Figures 3A,B). Further confirmation of Pb in the light precipitate was validated by the EDS analysis (data not shown).

### FT-IR Analysis

**FIGURE 4:**
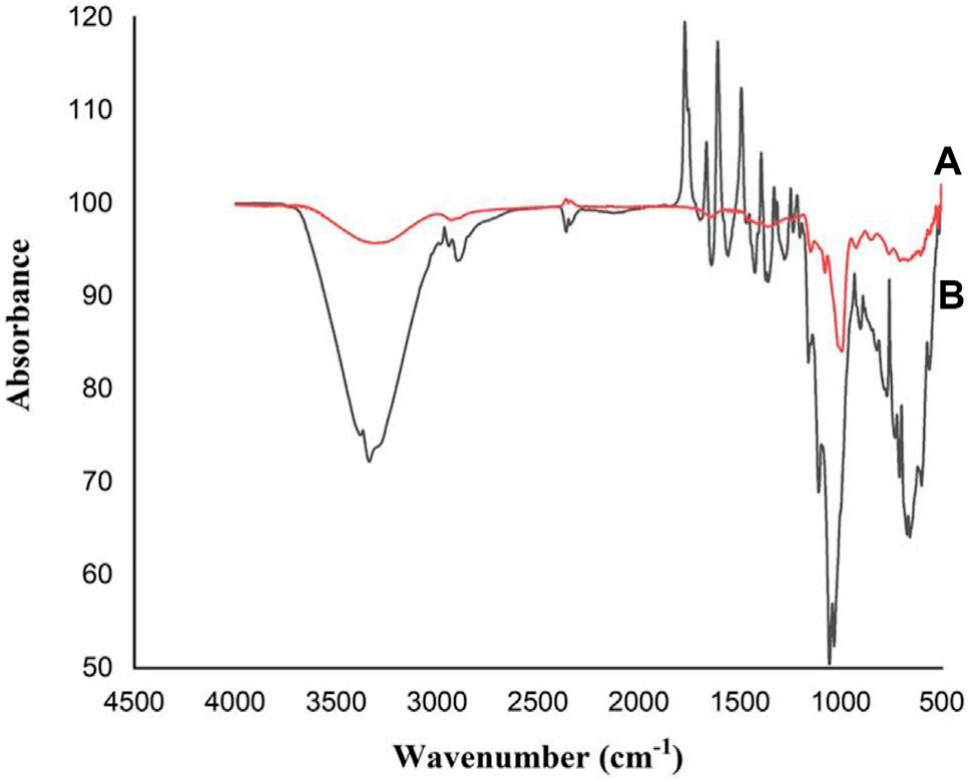
FT–IR or Fourier transform infrared spectrums of *L. acidophilus* KLDS1.1003 free and MRS-based encapsulated with Pb (60 mg/L): **(A)** Free *L. acidophilus* KLDS1.1003 biomass; and **(B)** MRS-based encapsulated *L. acidophilus* KLDS1.1003 biomass.

The FT-IR spectrums of free and MRS-based encapsulated *L. acidophilus* KLDS1.1003 and treated with Pb (60 mg/L) are shown in Figure 4. The noticeable shift to a substantial wave number at 3,550–3,100 cm^−1^ might be owing to the (−OH) group of starch and Pb interaction, and the desertion of the peak at 18,200–1,720, 1,680–1,660, 1,620–1,590, 1,520–1,480, 1,400–1,390, 1,250–1,240, 1,220–1,210, 1,170–938, and 926–541 cm^−1^ pointed out that the O−H, P=O or C−O broadening of polysaccharide vibrations participated in the Pb biosorption function of these polymeric materials. Meanwhile, a C=O elongating vibration of carboxylic acid was detected in the 2,370–2,320 cm^−1^ region. Similarly, a strong modification at 2,370–2,320 cm^−1^ wave number might be because of the Pb interaction with -OH group of alcohol-phenol and the –NH group of amide, respectively. Additionally, the amine II band (1,040–978 cm^−1^) is linked with broadening the C−N peptide bond and NH in-plane bending mode. These elements could be combined with lead (Pb) by the key functional groups like hydroxyl, phosphoryl, carboxyl, amino and, and amide groups.

### Body Weight of Mice

**FIGURE 5:**
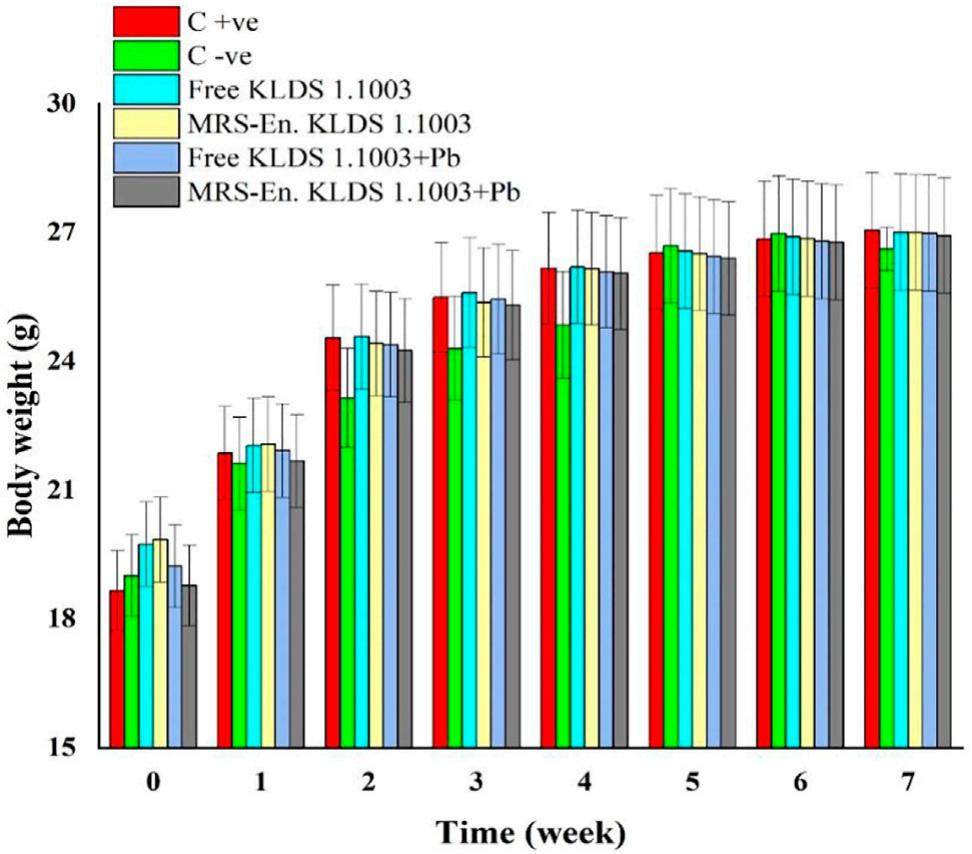
Assessment of the free and MRS-based microencapsulated *L. acidophilus* 1.1003 on mice’s body weights with lead fed in drinking water throughout the experimentation period of 7-weeks. Values aremean ± standard deviation (SD).

Keeping in view of the lead (Pb) biosorption capacities and tolerance of the lactic acid bacterial strains, *L. acidophilus* KLDS 1.1003 was selected as the best candidate for assessing its potentiality against lead toxicity in the forms of microencapsulated and free or nonencapsulated bacteria. During the experimentation period of 7 weeks, the weight of each mouse was observed, and it was found that there was no significant difference (*p* > 0.05) among all the studied groups. As shown in Figure 5, maximum bodyweight was witnessed in C +ve 27.05 ± 0.1342 g succeeded by 27.00 ± 0.13 g in the MRS based-microencapsulated KLDS 1.1003+Pb group, contrary to the C −ve group (26.62 ± 0.51 g) at 7th week of the study.

### Lead (Pb) Concentrations in the Kidneys, Liver and Blood

**FIGURE 6:**
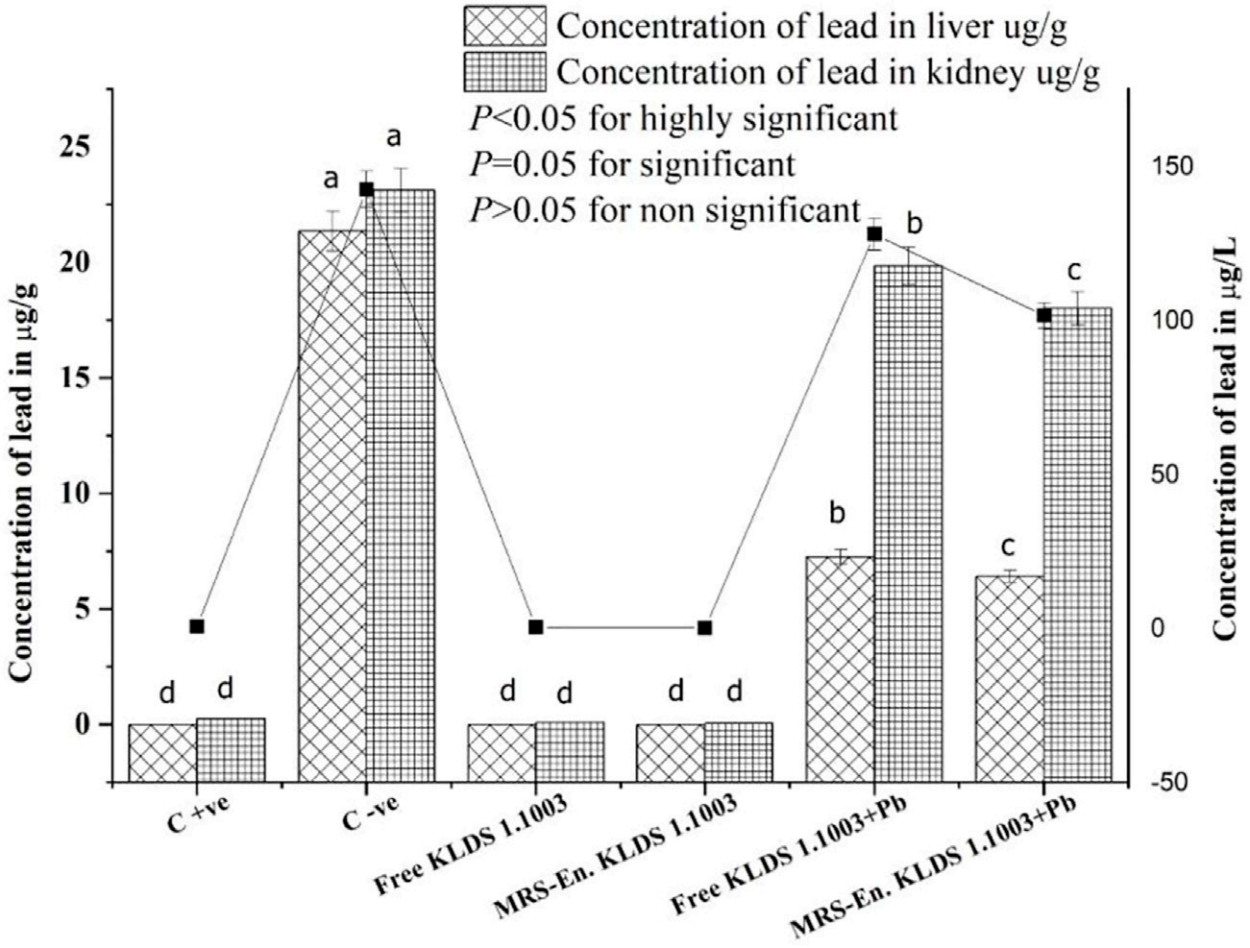
Influence of feeding free and MRS-based encapsulated *L. acidophilus* 1.1003 on the levels of Pb in the blood (µg/L) of mice presented with a solid line, in the kidney (µg/g) and liver (µg/g) of mice presented with bar with lead nitrate in drinking water. The significant difference (*p* < 0.05) is shown by letters. Values are mean ± standard deviation (SD).

According to Figure 6, among all the studied groups, the groups which were non-orally exposed to Pb, the Pb concentrations in the liver, kidneys, and blood were suggestively smaller than the orally exposed lead group (*p* < 0.05). The reduction of Pb levels in the blood and kidneys of the lead dose groups was significantly different from the observed results of the C−ve group (*p* < 0.05). Compared to the C−ve groups, free and MRS microencapsulated *L. acidophilus* KLDS 1.1003 treatments showed an expressive decrease of the Pb concentrations in tissues and blood. The Pb contents in the C +ve group tissues, free and MRS encapsulated KLDS 1.1003-only groups were very low in the liver and kidneys 0.25 ± 0.010 µg/g, 0.09 ± 0.003 µg/g, and 0.06 ± 0.002 µg/g respectively, as shown in Figure 6. As compared to the orally exposed C −ve (21.36 ± 0.872 µg/g in the liver 23.13 ± 0.944 µg/g in the kidney), free KLDS 1.1003+lead (7.27 ± 0.296 µg/g in liver 19.86 ± 0.810 µg/g in the kidney) and MRS encapsulated KLDS 1.1003+lead (6.42 ± 0.262 µg/g in the liver 18.02 ± 0.735 µg/g in the kidney) significantly (*p* < 0.05) reduced the lead (Pb) contents in the kidney and liver tissues of the studied mice. Concentrations of Pb in mice blood (µg/L) are provided in Figure 6. Contrary to the C −ve group (142.5 ± 5.817 µg/L), MRS encapsulated *L. acidophilus* KLDS 1.1003+lead showed a considerable Pb content reduction in tissues and blood (101.47 ± 4.142 µg/L) by protecting against the toxic effects of Pb in blood and tissues.

### Essential Elements (Ca, Zn, Fe, and Mg) Quantities in the Kidneys and Liver

During the study, variations in the concentration of essential elements (Ca, Mg, Fe, and Zn) in the kidneys and liver were observed due to the Pb exposure. These values are presented in (Table 4). Except for Zn, all other elemental variations in the kidneys and liver were significantly inverted in the free and encapsulated KLDS 1.1003+lead groups (*p* < 0.05). Nonetheless, compared to the C −ve group, a significant increase of the metal levels in the livers of all the other groups was observed.

**TABLE 4:** Effects of nonencapsulated and MRS-based encapsulated *L*. *acidophilus* 1.1003 on metal levels in the livers and kidneys of mice.

GroupLiver	Zn	Ca	Mg	Fe
C +ve	27.31 ± 1.00	300.05 ± 1.23^d^	231.11 ± 0.08^c^	79.98 ± 3.92^a^
C−ve	28.08 ± 1.41	241.70 ± 1.33^e^	209.22 ± 2.26^f^	34.04 ± 1.72^e^
Free *L. acidophilus* KLDS 1.1003	26.27 ± 1.31	321.21 ± 2.03^b^	230.36 ± 0.05^d^	69.53 ± 3.47^b^
MRS-based encapsulated *L. acidophilus* KLDS 1.1003	25.05 ± 1.25	347.23 ± 4.29^a^	248.26 ± 1.36^a^	72.01 ± 3.60^a, b^
Free *L. acidophilus* KLDS 1.1003 + lead	25.22 ± 1.26	296.64 ± 3.02^f^	219.24 ± 4.13^e^	42.09 ± 2.10^d^
MRS-based encapsulated *L. acidophilus* KLDS 1.1003 +lead	24.12 ± 1.20	303.09 ± 1.52^c^	237.31 ± 2.02^b^	46.94 ± 2.34^c^
**Kidney**				
C +ve	16.92 ± 0.84	815.22 ± 3.22^d^	178.98 ± 2.11^f^	52.31 ± 0.61^d^
C −ve	22.32 ± 1.11	824.27 ± 3.09^c^	202.62 ± 0.03^d^	48.29 ± 2.41^e^
Free *L. acidophilus* KLDS 1.1003	20.28 ± 1.01	802.02 ± 2.02^f^	210.62 ± 0.11^b^	59.14 ± 2.95^b^
MRS-based encapsulated *L. acidophilus* KLDS 1.1003	20.31 ± 1.01	806.08 ± 1.28^e^	223.20 ± 1.53^a^	60.52 ± 3.02^a, b^
Free *L. acidophilus* KLDS 1.1003 + lead	20.77 ± 1.03	832.09 ± 2.11^b^	193.07 ± 2.23^e^	53.30 ± 2.66^c, d^
MRS-based encapsulated *L. acidophilus* KLDS 1.1003 +lead	20.93 ± 1.04	837.26 ± 3.23^a^	204.21 ± 1.04^c^	54.87 ± 2.74^c^

Note: Data are expressed as mean ± standard deviation.

According to the Tukey means post comparison test, different letters within different rows designate the significant difference.

### GSH, GPx, SOD, CAT and MDA in Renal and Hepatic Tissues

**FIGURE 7:**
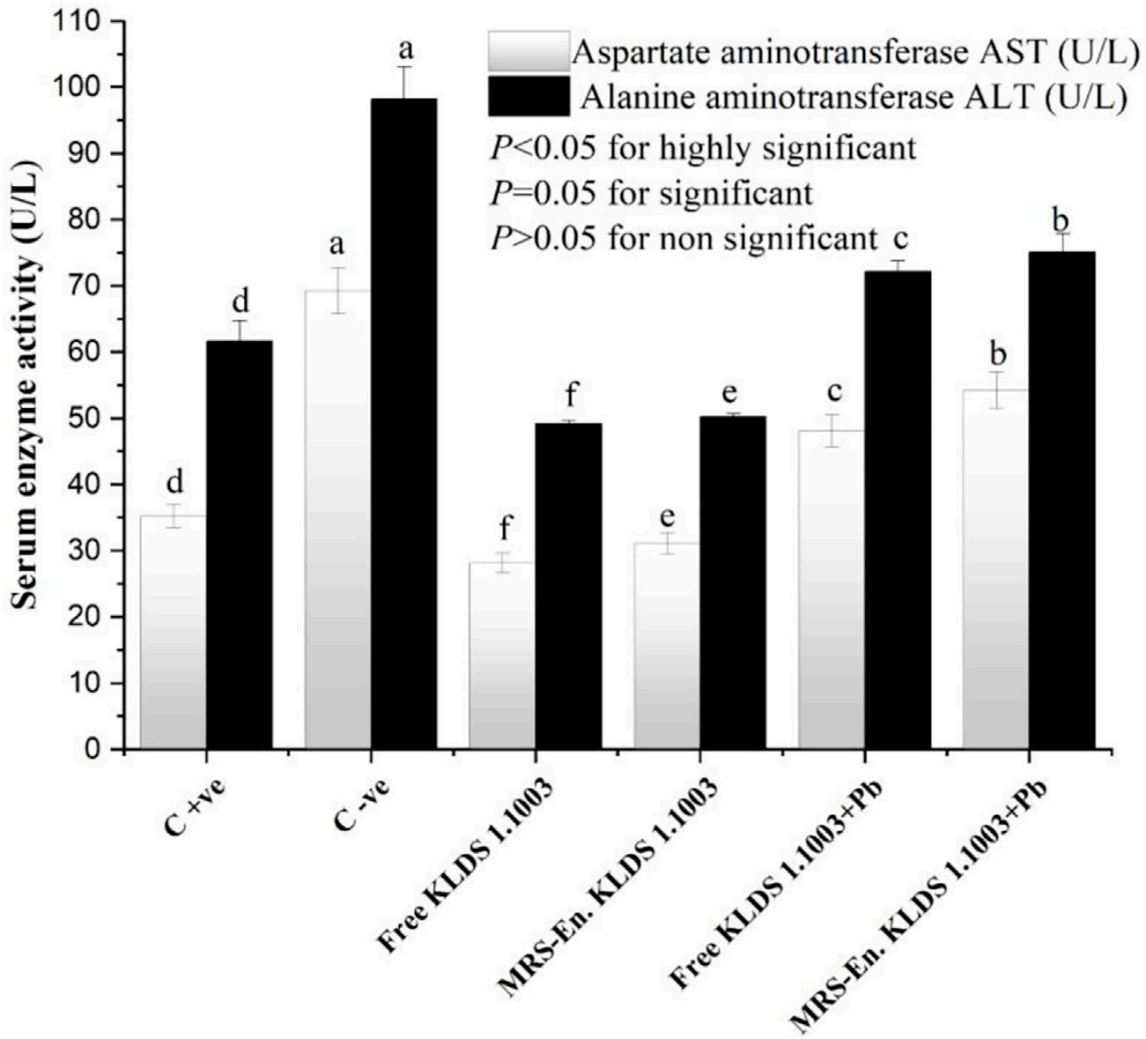
Effects of free and MRS-based microencapsulated *L. acidophilus* KLDS 1.1003 on oxidative stress induced by chronic Pb exposure on the activity of marker enzymes such as aspartate aminotransferase (AST) and alanine aminotransferase (ALT) in the serum of mice. Values are mean ± SD.

The capacity levels of anti-oxidating enzymes in the mice kidneys and liver have been shown in Table 5. The levels of GSH, SOD, and CAT were significantly decreased when the mice were exposed to lead (Pb) belonging to both non-orally and orally exposed groups. This reduction was due to the chronic exposure of these mice to Pb, while on the other hand, MDA and GSH-Px levels were increased simultaneously. Integration of the MRS-based microencapsulated *L. acidophilus* KLDS 1.1003 treatment effectually maximized the CAT levels in the liver (223.89 ± 1.41 U/mgprot as compared to the C −ve group 112.62 ± 2.03 U/mgprot). SOD and GSH were increased, whereas GSH–Px and MDA levels were significantly decreased (*p* < 0.05). As a whole, both the microencapsulated and free forms of KLDS 1.1003 imparted substantially protective properties against the chronic toxicity of Pb and helped in restoring and enhancing the antioxidant capacity (*p* < 0.05). Similarly, the variations in the levels of these parameters were protected significantly in the oral introduction of co-treatment with free and MRS encapsulated KLDS 1.1003+lead groups. In the same manner, the MDA and GSH–Px levels were increased in kidneys after chronic exposure to Pb while the activities of SOD, GSH, and CAT enzymes were improved considerably. Marker enzymes, i.e., AST and ALT activities in the mice serum, have been presented in Figure 7.

**TABLE 5:** Effects of free and MRS-based encapsulated *L. acidophilus* 1.1003 on oxidative stress induced by chronic Pb exposure, differences of antioxidant capability in the kidney and liver.

GroupLiver	SOD (U/mgprot)	MDA (nmol/gprot)	GSH (umol/mgprot)	GSH-PX (U/mgprot)	CAT (U/mgprot)
C +ve	45.52 ± 0.62^c^	0.41 ± 0.01^f^	4.47 ± 0.11^a^	86.64 ± 2.41^a^	318.42 ± 0.38^a^
C −ve	32.35 ± 0.06^f^	0.52 ± 0.03^a^	1.33 ± 0.13^f^	46.15 ± 2.32^f^	111.18 ± 1.01^f^
Free *L. acidophilus* KLDS 1.1003	46.66 ± 0.38^b^	0.45 ± 0.03^e^	3.36 ± 0.53^d^	74.25 ± 1.56^c^	173.52 ± 0.29^d^
Free *L. acidophilus* KLDS 1.1003 + lead	34.12 ± 0.04^e^	0.47 ± 0.01^d^	2.42 ± 0.09^e^	53.48 ± 1.59^e^	168.12 ± 0.21^e^
MRS-based encapsulated *L. acidophilus* KLDS 1.1003	46.76 ± 0.07^a^	0.46 ± 0.01^c^	3.75 ± 0.10^b^	76.41 ± 0.36^b^	223.89 ± 1.41^b^
MRS-based encapsulated *L. acidophilus* KLDS 1.1003 +lead	35.19 ± 0.01^d^	0.51 ± 0.02^b^	3.61 ± 0.07^c^	63.72 ± 1.88^d^	176.62 ± 0.40^c^
**Kidney**					
C +ve	36.96 ± 0.22^c^	0.85 ± 0.06^b^	3.38 ± 0.07^b^	63.19 ± 2.69^b^	76.89 ± 0.12^a^
C −ve	29.44 ± 0.23^f^	1.16 ± 0.03^a^	1.47 ± 0.21^f^	85.71 ± 2.41^a^	55.99 ± 0.21^f^
Free *L. acidophilus* KLDS 1.1003	35.22 ± 0.09^d^	0.73 ± 0.02^e^	3.57 ± 0.05^a^	49.53 ± 2.61^e^	65.15 ± 0.14^b^
Free *L. acidophilus* KLDS 1.1003 + lead	34.24 ± 0.06^e^	0.84 ± 0.02^c^	2.65 ± 0.13^d^	59.01 ± 0.44^c^	58.83 ± 0.20^d^
MRS-based encapsulated *L. acidophilus* KLDS 1.1003	38.69 ± 0.15^a^	0.70 ± 0.01^f^	2.88 ± 0.08^c^	49.57 ± 1.83^e^	62.33 ± 0.51^cd^
MRS-based encapsulated *L. acidophilus* KLDS 1.1003 +lead	37.70 ± 0.07^b^	0.80 ± 0.03^d^	2.37 ± 0.11^e^	54.15 ± 1.92^d^	63.08 ± 0.62^c^

Note: Data are expressed as mean ± standard deviation.

According to the Tukey means post comparison test, different letters within different rows designate the significant difference.

### Histopathological Evaluation

**FIGURE 8:**
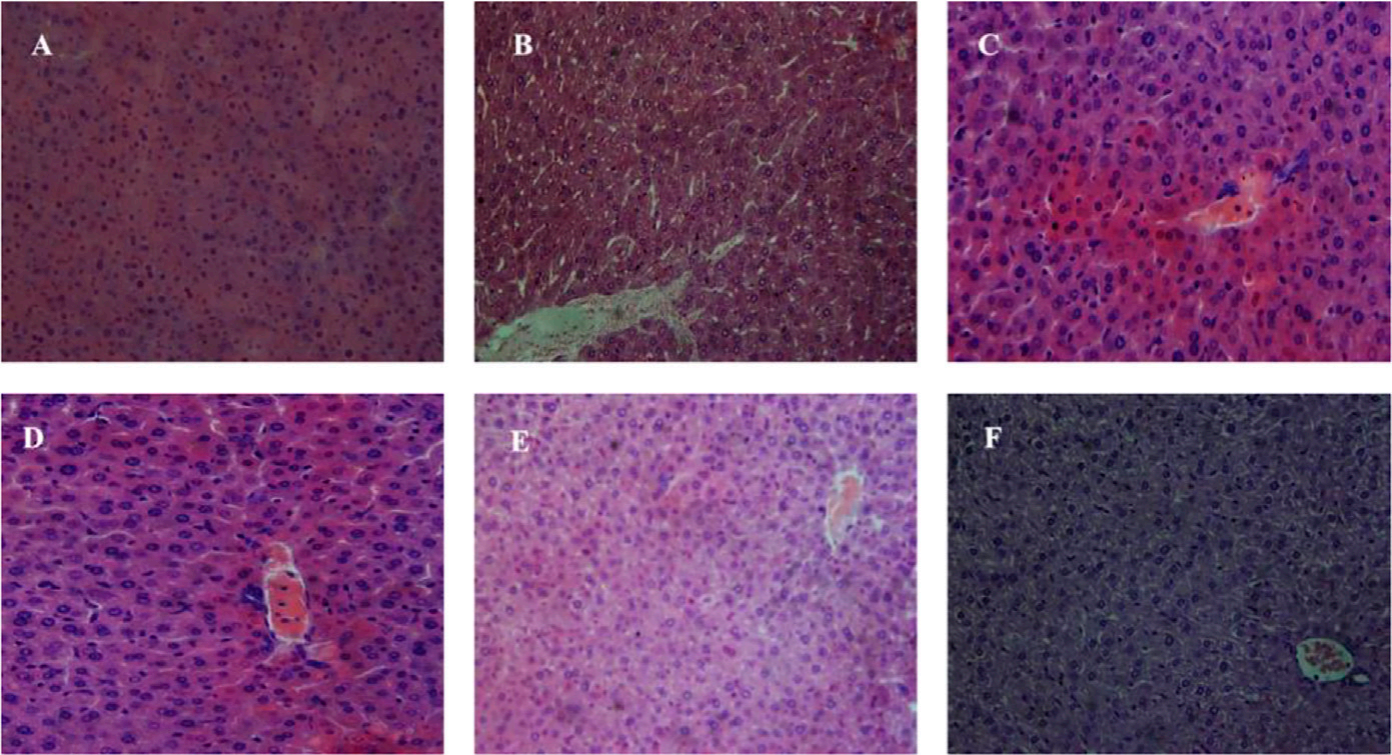
Micrographs express the hepatic (liver) tissues of the mice hematoxylin–eosin (H&E staining; magnifications ×400). **(A)** C +ve, **(B)** C −ve, **(C)** *L. acidophilus* KLDS 1.1003 only, **(D)** MRS-based encapsulated *L. acidophilus* KLDS 1.1003 only, **(E)** *L. acidophilus* KLDS 1.1003 plus lead, **(F)** MRS-based encapsulated *L. acidophilus* KLDS 1.1003 plus lead.

**FIGURE 9:**
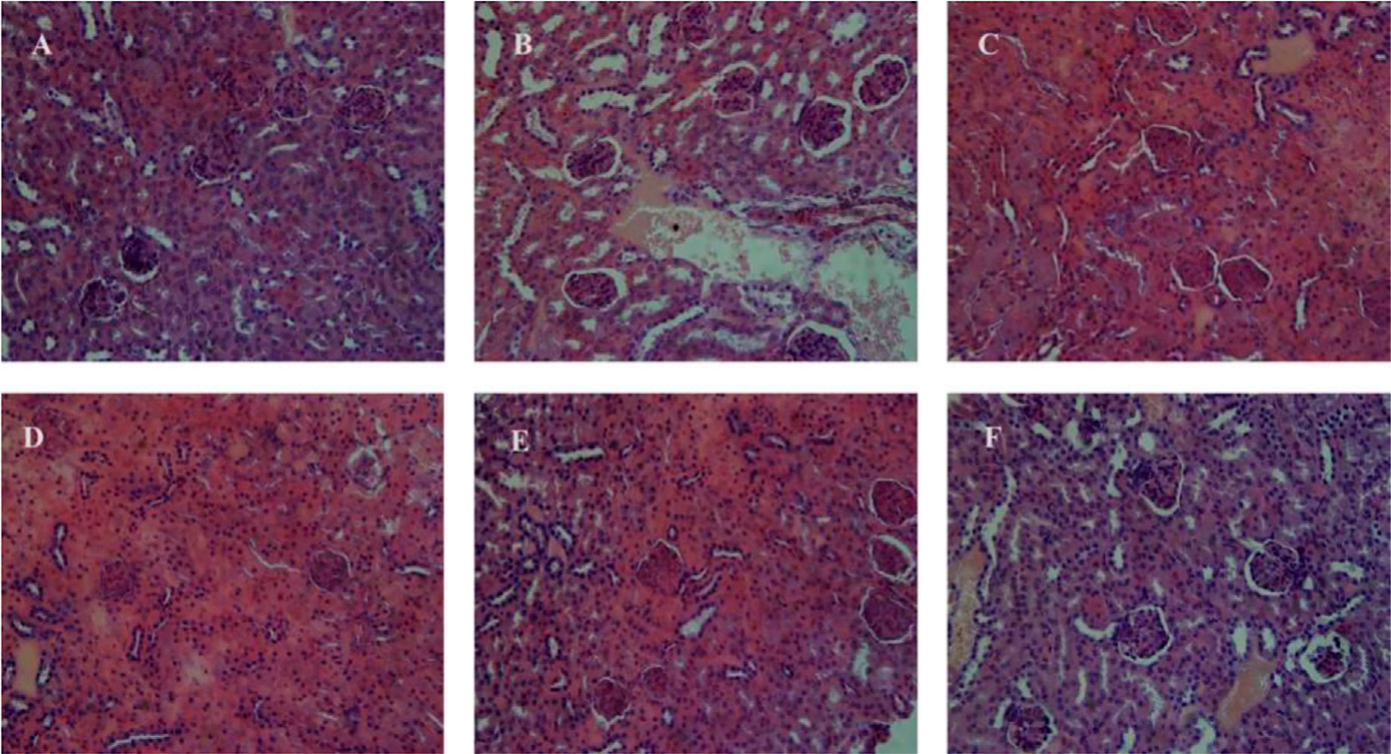
Micrographs express the renal (kidney) tissues of the mice hematoxylin–eosin (H&E staining; magnifications ×400). **(A)** C +ve, **(B)** C −ve, **(C)** *L. acidophilus* KLDS 1.1003 only, **(D)** MRS-based encapsulated *L. acidophilus* KLDS 1.1003 only, **(E)** *L. acidophilus* KLDS 1.1003 plus lead, **(F)** MRS-based encapsulated *L. acidophilus* KLDS 1.1003 plus lead.

Histopathological studies showed that the mice tissues were severely harmed after chronic Pb exposure. The representative micrographs of the liver and kidney tissue samples of all groups are displayed in Figures 8, 9. The hepatic damages were significantly relieved when treated with MRS microencapsulated and free KLDS 1.003 plus Pb (Figure 8). Histological alterations were produced in the liver after chronic exposure to Pb. These alterations were comprised of cytoplasmic vacuolization, chromatin condensation, and injury of intact liver plates. Similarly, histopathological analyses of the liver in the C −ve group Figure 8B displayed microvascular steatosis vacuolar degeneration and enlarged hepatocytes. Whereas the control Figure 8A did not show any obvious histopathological changes. At the same time, certain hepatocytes were observed, which swelled exceptionally. The protective effect against these swelling injuries is clear in groups with *L. acidophilus* KLDS 1.1003 only Figure 8C and MRS-based encapsulated *L. acidophilus* KLDS 1.1003 Figure 8D. But *L. acidophilus* KLDS 1.1003 plus lead Figure 8E and MRS-based encapsulated *L. acidophilus* KLDS 1.1003 plus lead Figure 8F showed better protective effects against the identical injuries in the liver.

Figure 9 shows the micrographs of kidney tissues, which clearly show the enlargement of glomeruli, cloudy swelling and necrosis of tubules in C −ve treated groups, as compared to the control group. These renal damages were significantly relieved by free, and MRS encapsulated KLDS 1.1003+ lead treatments. Figure 9A shows the histomorphology of the kidney tissues of C +ve groups, which is apparently normal. It was noticed that the glomeruli were hyperemic (glomeruli with bigger volume) in C −ve group, as these can be seen in Figure 9B. In addition, the swelling was also seen in the tubular epithelial cells of the renal tissues, and the missing glomeruli was also observed (Figures 9B,D,F). Some symptoms of the renal oxidative stress like swollen tubular epithelial cells (ST), glomerular hyperemic (GH), inflammatory cells (I), and granular degeneration (GD) were ameliorated (Figures 9C–F) in all treated groups comparing to the C −ve group (Figure 9B).

No significant variations or differences were found in the hepatic and renal morphology of the free and MRS-based microencapsulated KLDS 1.003 treated groups after comparing with that of the C +ve group.

## Discussion

Lead (Pb) is a heavy metal pollutant that has no constructive biological role in the body, and it is a pestilent pollutant having ubiquitous existence in the environment. It can harm the host by inducing oxidative stress. Several research reports evidentially reported that lactic acid bacteria have the capability to sequester Pb metal ions as well as cope with the oxidative stress induced by its acute and chronic toxicity (Zhai et al., 2013, 2014; Li B. et al., 2017; Muhammad et al., 2018). Several properties should be taken into consideration during the screening process of specific LAB strains tackling Pb toxicity. The first and foremost criterion is the ability of LAB strains to exhibit higher Pb binding capability, which facilitates these strains to bind Pb before the host gets it through intestinal absorption. Secondly, these LAB strains should avoid toxicity by showing resistance to Pb ions and averting their noxiousness (Zhai et al., 2015; Allam et al., 2018).

Similarly, the higher viability of screened strains in highly concentrated bile and stomach acids is pivotally important to remove Pb from the gastrointestinal (San Keskin et al., 2018). As far as the present study is concerned, among the six tested *L. acidophilus* KLDS strains, a remarkably best tolerance and Pb binding aptitude have been shown by *L. acidophilus* KLDS1.1003 (Table 2). In addition, the Pb biosorption mechanism of this strain was also investigated. Moreover, maize-resistant starch-based microencapsulated *L. acidophilus* KLDS1.1003 not only showed significant resistance against the simulated gastrointestinal conditions, but also improved the Pb binding properties too (Figure 2). Considering these properties, *L. acidophilus* KLDS 1.1003 was selected as a free and microencapsulated potential LAB strain to study the *in vivo* assays further.

Different biosorption mechanisms to sequester heavy metals have been proposed depending upon the differences in the structure of bacterial species. These mechanisms include chelation and micro precipitation, complexation, ion exchange, and adsorption (Monachese et al., 2012). The contents like proteins, polysaccharides, teichoic acids, and peptidoglycans are present in the cell walls of gram-positive bacteria (Passot et al., 2015). The bacterial wall contents contain negatively charged functional groups, which act as the primary sites of metal ion sorption on the bacterial surfaces (Mishra et al., 2013; Yadav et al., 2014). The phosphate and carboxyl groups present in peptidoglycans and teichoic acid contents of the bacterial cell surface are the potential metal ion binding sites (Kazy et al., 2009). As it is obvious in the given SEM micrographs (Figure 3) that there is an even localization of the added Pb metal ion on the cell surface of bacteria, which confirms the theory as mentioned above. In previous research, a similar passive physicochemical phenomenon of heavy metal binding has been reported in *L. bulgaricus* (Li B. et al., 2017) and *L. mesenteroides* (Yi et al., 2017), explaining the Pb adsorption mechanism.

In our study, free and microencapsulated *L. acidophilus* were used as biosorbents. The chemical modification and surface characterization of these biosorbents due to the biosorption of metal ions were examined by the FTIR analysis. The involvement and identification of functional groups of free and microencapsulated bacterial biomass during the biosorption process was observed by this analysis. The highest observed spectral differences in the appearance of the spectrum are shown in Figure 4. Due to the presence of a large number of peaks, the FTIR spectra of free and microencapsulated *L. acidophilus* after biosorption indicated a complex nature of the biomass. The difference of biosorption was witnessed in the starch-based encapsulated and free bacterial biomass due to the increase or decrease in peak intensities, appearance of new peaks and shifting of the spectral bands. The functional groups are present in the structure of macromolecules like lipids, proteins, and polysaccharides.

Additionally, the bacterial cell wall offers many functional binding sites, such as sulfonates or phosphates, amines, carboxyl, and hydroxyl groups as well as, the shifting and broadening of the peaks were detected due to the interaction of metal ions with starch as compared to free bacterial biomass of the studied strains. The speculation that the carboxyl, hydroxyl, amino, amide, and phosphoryl functional groups of biological macromolecules have the ability to bind Pb ions through electrostatic attraction (physical adsorption), complexation, and ion exchange. Similarly, biopolymers like polysaccharides, teichoic acid, S-layer proteins, and fatty acids follow the mentioned mechanism for sequestering the Pb ions. These findings are consistently supported by the previous statements that the amide, hydroxyl, and carboxyl functional groups played a key role in uptaking the Pb (Afraz et al., 2020; Massoud et al., 2020).

Ren et al. also conducted a study related to the biosorption of Pb by using the *Bacillus* sp. PZ-1, and it supports the findings of our present research work (Ren et al., 2015). Moreover, the glucose pyranose rings showed skeletal mode vibrations due to which the complex vibrational modes of starches were shown at low wavenumbers (below 900 cm^−1^) (Soto et al., 2015, 2016). In addition, the active involvement of various carboxylic, hydroxyl, carbonyl, amine, and phosphate functional groups was also observed in starch-based microencapsulated biomass to sequester the metal ions of Pb (Bueno et al., 2008; Ma et al., 2015; Mohapatra et al., 2019).

In order to imitate the chronic or prolonged lead (Pb) contact, it was orally supplied in drinking water. The concentration of Pb was taken as 100 mg/L to demonstrate the normal human and animal pertinent Pb ecological exposure in contaminated zones. This concentration was chosen, conferring the human and animal levels of Pb ingestion and body weight intensities (Fan et al., 2008). Herewith, we have investigated that microencapsulated *L. acidophilus* KLDS 1.1003 developed a superior capability to chelate Pb. The enhancement in capability might be due to the maize-resistant starch, which provided substantial protection to bacteria against *in vivo* lead toxicity and also helped in the chelation of Pb ions, resulting in an ultimate decrease in lead concentrations in blood as well as in tissues (Figure 6.)

Similarly, KLDS 1.1003 also prohibited variations in the levels and activities of GSH, GSH-Px, MDA, and SOD in blood and tissues supporting antioxidant systems against induced oxidative stress. Our observations revealed that the free and microencapsulate *L. acidophilus* KLDS 1.1003 had affected the lead toxicity mechanism quite significantly by providing relief against lead-induced chronic toxicity. Lipid peroxidation and oxidative stress processes are accelerated as the MDA levels are increased, which resultantly disturb the SOD, DSH-Px, and GSH enzyme activities and eventually influence the antioxidant defense system of the body (Li B. et al., 2017; Muhammad et al., 2018).

Tian et al. and Muhammad et al. investigated the role of *L. plantarum* CCFM8661 on supporting antioxidant mechanisms by improving the enzymatic activity, plummeting lead concentrations in blood and tissues and, appeasing oxidative stress (Tian et al., 2015; Muhammad et al., 2018; Kinoshita et al., 2020). Li B. et al. (2017) also publicized that *L. bulgaricus* decreased the Pb-induced oxidative stress and played a protective role by facilitating the increased levels of antioxidants like T-SOD, GSH–Px, and GSH and the reduction of MDA and lipid peroxide levels in the liver and kidneys. Mitochondrial dysfunction and energy metabolism impairment are lured by the GSH reduction (Pereira and Oliveira, 2000; Patel et al., 2017). It has also been observed that *lactobacilli* have a quicker and steadier biosorption ability to sequester lead *in vitro* with minimum chances of smooth desorption (Schut et al., 2011; Lin et al., 2019).

Furthermore, the loss of Fe, Zn, Ca, and Mg (crucially essential) metals from the mice tissues was not done by the KLDS 1.1003 itself did (Table 4). Even a certain increase was observed in several vital elements, e.g., calcium (Ca) in the liver and iron (Fe) in the kidneys. Our study results indicated that the MRS-based microencapsulated KLDS 1.1003 therapy group contained a better potential to bind and defecate higher quantities of Pb from the animal body than free KLDS 1.003 groups. Some other *lactobacilli* also showed the corresponding observations by confining the heavy metal’s absorption within the intestine of the mice and then excreting them through feces (Jomova and Valko, 2011; Zhai et al., 2013, 2014). Huang et al. (2011;2015) and Liu et al. (2018) also investigated and concluded that the structure of modified starches has the potential to confiscate Pb^2+^ (lead) and Hg^2+^ (mercury) ions (Huang et al., 2011, 2015; Liu et al., 2018). Usually, the prominent respective adsorption capabilities of modified starches are 123.2 and 131.2 mg/g for Pb^2+^ and Hg^2+^. In addition, San Keskin et al. (2018) also reported higher efficiencies (70 ± 0.2, 58 ± 1.4 and 82 ± 0.8%) to remove Ni and Cr heavy metal ions from 30 mg L^−1^ concentrated solutions by using the *Lysinibacillus* sp. microencapsulated with cyclodextrin fiber (San Keskin et al., 2018).

According to several studies, there has been a possible relation between oxidative stress and disrupted homeostasis by heavy metal ions (Odewabi and Ekor, 2017; Galaris et al., 2019; Yang et al., 2020). Changes in AST/ALT levels are frequently used to study liver pathology as AST/ALT is an imperatively vital biochemical indicator (Soliman et al., 2020; Aljobaily et al., 2021). The cell plasma of the liver is the main existing place of ALT. As the liver-cell membrane permeability changes, the ALT levels are increased in the blood with the lower damage of the liver cells. In contrast, the escalation of AST levels in the blood indicate severely damaged liver cells, as can be seen in the data presented in Figure 7. Keeping in view of the above discussion, it could be conclusively stated that the higher the AST/ALT ratios, the worst damage will be experienced to liver cells. According to several recently conducted studies, it could be concluded that the LAB (*L. bulgaricus* and *L. plantarum*) treatments can potentially intensify the ALT and AST levels. While, if the LAB’s are microencapsulated with modified starches like MRS then, the heavy metal absorbing ability of probiotic lactic acid bacteria can be increased (Li B. et al., 2017; Liu et al., 2018; Muhammad et al., 2018; San Keskin et al., 2018; Mohammadian et al., 2020). And these prebiotic/probiotic combinations can be taken as a natural biosorbing remedy to tackle the induced oxidative stress by chronic toxicity of Pb and other toxic heavy metals.

## Conclusion

*L. acidophilus* KLDS1.1003 showed great *in vitro* and *in vivo* capacities with respect to the tolerance and biosorption of Pb and relieved against induced oxidative stress. The main functional groups, i.e., hydroxyl, carboxyl, phosphoryl, amino and, amide groups in bacterial cell walls and polysaccharide structure, played a key and efficient role in the complexation and adsorption Pb ions. Free and MRS-based microencapsulated KLDS 1.0344 not only improved the antioxidant index and inhibited changes in blood and serum enzyme concentrations and also relieved the Pb-induced renal and hepatic pathological damages. *L. acidophilus* KLDS1.1003 in both forms facilitated the detoxification process of Pb *in vivo*. It conserved the antioxidant defense system of the Pb exposed animal by maintaining the activities and concentrations of GSH, GPx, SOD, CAT, and MDA in renal and hepatic tissues and ALT/AST ratios in blood and serum. Conclusively, a symbiotic combination of prebiotics/probiotics in Pb binding, bioquenching, damage repairing capacities of orally administered MRS microencapsulated and free *L. acidophilus* KLDS 1.1003 bacteria against inflammation and induced oxidative stress due to chronic exposure of Pb make them ideal and natural remedying candidates tackling heavy metal lethality.

## Data Availability

The raw data supporting the conclusions of this article will be made available by the authors, without undue reservation.
